# Protocol to maintain ECT in COVID-19 pandemic

**DOI:** 10.1192/j.eurpsy.2021.1773

**Published:** 2021-08-13

**Authors:** M. Martínez-Roig, J. Arilla-Aguilella, A. Arriola-Segura, R. Rolando-Martinez

**Affiliations:** Psychiatry, Hospital Royo Villanova, Zaragoza, Spain

**Keywords:** Affective disorders, ECT, COVID-19

## Abstract

**Introduction:**

Electroconvulsive therapy is a highly effective treatment for severe psychopharmacological resistant patients but it is also a procedure that involves open airway management and has been considered as an aerosol generating procedure. The COVID-19 pandemic, has resulted in reduction in ECT services internationally. The COVID-19 pandemic has dramatically and rapidly transformed hospitals in heavily affected areas, decreasing mental health services. The need to locate critical patients in spaces intended for anesthesia, where we usually administered ECT, has forced us to decrease the number of procedures and be highly selective. In the same way, continuation and maintenance ECT (m-ECT) have also been dramatically reduced. The risk of contagion urged us to develop a protocol involving other areas of the hospital

**Objectives:**

To create a safe circuit from admission to the hospital to the ECT including emergency room and psychiatric Ward

**Methods:**

Review of the tliterature and published protocols Workshops with Preventive Medicine, Anaesthesia and Emergency Service to elaborate a protocol Submission of the protocol to Management of the Hospital

**Results:**

The protocol (Figure 1) began with the screening for COVID-19 in every patient. If the PCR was (+) the patient was not excluded. We moved treatment from the PACU into the OR and if a patient tested positive It was determined that the ECT was administered in the OR

**Figure:**
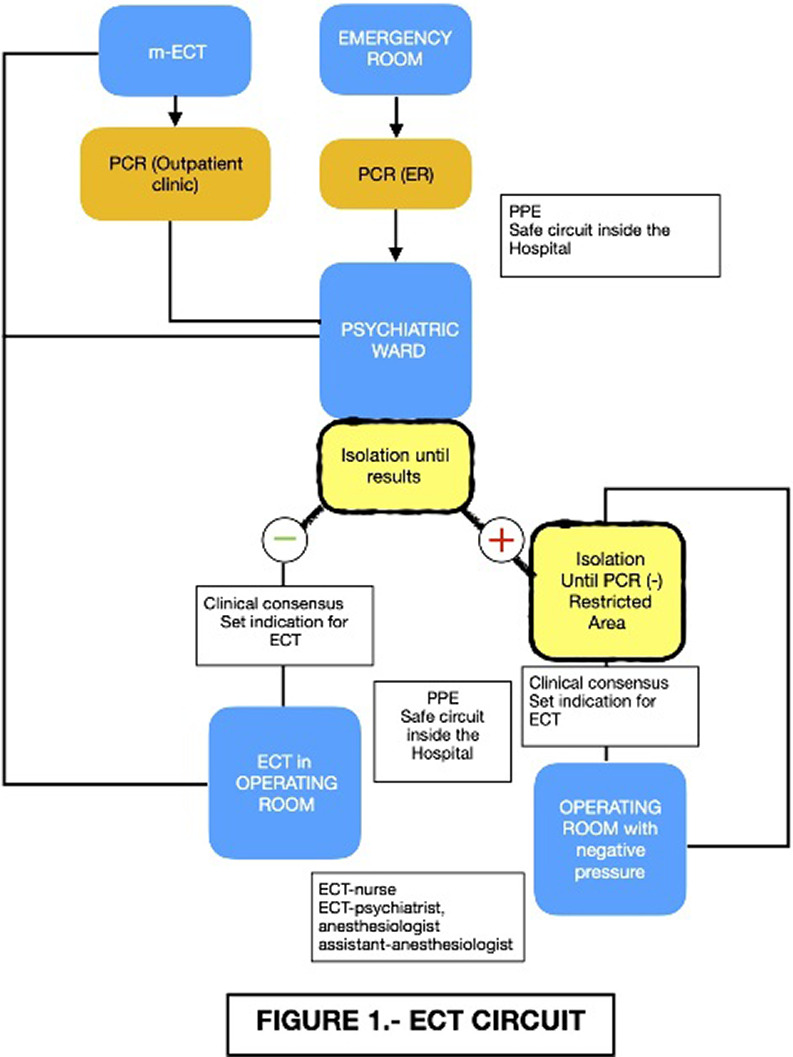

That was provided with negative pressure. Circuits were established within the Psychiatric Ward and in the areas of the hospital involved to reduce risks and patients remained isolated until negative test was confirmed The number of persons present in the treatment room was kept to the absolute minimum required and appropriate personal protective equipment was used, as prescribed by the WHO

**Conclusions:**

We must keep in mind treating the most vulnerable of our patients. ECT should be seen as an essential medical procedure and made available

**Disclosure:**

No significant relationships.

